# Management of Primary Biliary Cholangitis: Current Treatment and Future Perspectives

**DOI:** 10.5152/tjg.2023.22239

**Published:** 2023-02-01

**Authors:** Romelia Barba Bernal, Bryan Ferrigno, Esli Medina Morales, Cristina M. Castro, Daniela Goyes, Hirsh Trivedi, Vilas R. Patwardhan, Alan Bonder

**Affiliations:** 1 Beth Israel Deaconess Medical Center, Boston, MA, USA; 2 Loyola Medicine, MacNeal Hospital, Berwyn, IL, USA

**Keywords:** Investigational therapies, primary biliary cholangitis, risk stratification, therapeutic advances

## Abstract

Primary biliary cholangitis is an autoimmune cholestatic liver disease characterized by progressive destruction of bile ducts, which can ultimately progress to chronic liver disease and cirrhosis. Ursodeoxycholic acid and obeticholic acid are the only 2 Food and Drug Administration (FDA)-approved medications for primary biliary cholangitis. Unfortunately, up to 40% of patients with primary biliary cholangitis have an incomplete response to ursodeoxycholic acid, warranting an essential need for additional therapeutics. Peroxisome proliferator-activated receptor agonists have shown promising data supporting their use as disease-modifying therapies. Fibroblast growth factor-19 agonists, farnesoid X receptor agonists, and nicotinamide adenine dinucleotide phosphate (NADPH) oxidase 3 inhibitors are additional agents under investigation as potential disease-modifying therapy. However, evidence supporting the use of certain novel therapies over others is sparse. There is a need for additional clinical trials as well as research aimed at the underlying pathophysiology of primary biliary cholangitis to discover additional therapeutic targets.

**Main Points**

- First-line treatment for primary biliary cholangitis (PBC) includes initiation of ursodeoxycholic acid (UDCA). Obeticholic acid (OCA) should be added if there is a sub-optimal response to UDCA, but OCA is contraindicated in patients with decompensated cirrhosis and compensated cirrhosis with evidence of portal hypertension.
- Up to 40% of patients with PBC have an incomplete response to UDCA, this patient population requires additional pharmacotherapies with the goal of inducing biochemical remission.
- Peroxisome proliferator-activated receptor agonists including bezafibrate, fenofibrate, and elafibranor have shown promise in inducing biochemical remission in PBC patients who are UDCA non-responders. Further studies assessing hard outcomes including mortality are needed.
- Additional investigational therapies including fibroblast growth factor-19 agonists, farnesoid X receptor agonists, and NADPH oxidase 3 inhibitors are being explored as potential disease-modifying therapies.

## Introduction

Primary biliary cholangitis (PBC) is an autoimmune and progressive form of cholestatic liver disease. It is characterized by T-cell-mediated destruction of primarily intra-lobular bile ducts. Progressive destruction of bile ducts leads to worsening cholestasis and subsequently chronic liver disease, which is the primary driver of morbidity and mortality in this patient population. If left untreated, the natural history of PBC results in an increased risk of developing cirrhosis.

Patients with PBC are predominantly female (>90%) and are typically diagnosed in the fourth or fifth decade of life. The diagnosis of PBC is suspected in women with fatigue, pruritus, abdominal pain, and/or jaundice in combination with abnormal liver function tests, primarily those with elevated alkaline phosphatase. The diagnostic criteria for PBC have been set forth by published practice guidelines. Primary biliary cholangitis can be diagnosed when 2 of 3 criteria are met: (1) evidence of cholestasis based on an elevated alkaline phosphatase, (2) presence of antimitochondrial antibodies (AMA) or other PBC-specific auto-antibodies (Sp100, GP210) if AMA is negative, and/or (3) histologic evidence of non-suppurative destructive cholangitis and destruction of interlobular bile ducts.^1^ Primary biliary cholangitis patients are at risk for concomitant extra-hepatic autoimmune diseases, including autoimmune thyroid disease, Sjogren’s disease, and systemic sclerosis, among others.^2^

Currently, there are only 2 Food and Drug Administration (FDA)-approved treatments for PBC, ursodeoxycholic acid (UDCA) and obeticholic acid (OCA). Ursodeoxycholic acid is considered first-line pharmacotherapy and is typically initiated at the time of diagnosis at a dose of 13-15 mg/kg/day. Unfortunately, approximately 40% of patients with PBC have an incomplete response at 1 year and carry a significantly worse prognosis in terms of progression to cirrhosis and overall mortality compared to responders.^1,3^ While criteria to assess response vary (Table 1), commonly used values are improvement in the alkaline phosphatase to <1.67 times the upper limit of normal after 12 months of UDCA therapy.^4^

Patients who fail to meet these criteria are deemed “non-responders,” and while they remain on UDCA going forward unless it is not tolerated, additional medications are needed with the goal of inducing biochemical remission.^5,6^

Furthermore, patients with PBC and advanced fibrosis have higher rates of mortality and morbidity even if they exhibit adequate biochemical response to UDCA, suggesting the need for more aggressive treatment regimens in those with fibrosis.^7^

Therefore, additional therapeutics for the management of PBC, particularly for those with advanced fibrosis and UDCA non-responders, are urgently needed. The purpose of this review is to discuss the current pharmacological management of PBC, as well as potential new therapeutics that are under investigation as disease-modifying therapies (DMTs).

## Current Primary Biliary Cholangitis Management

### *The Role of* Ursodeoxycholic Acid

**Figure 1.**
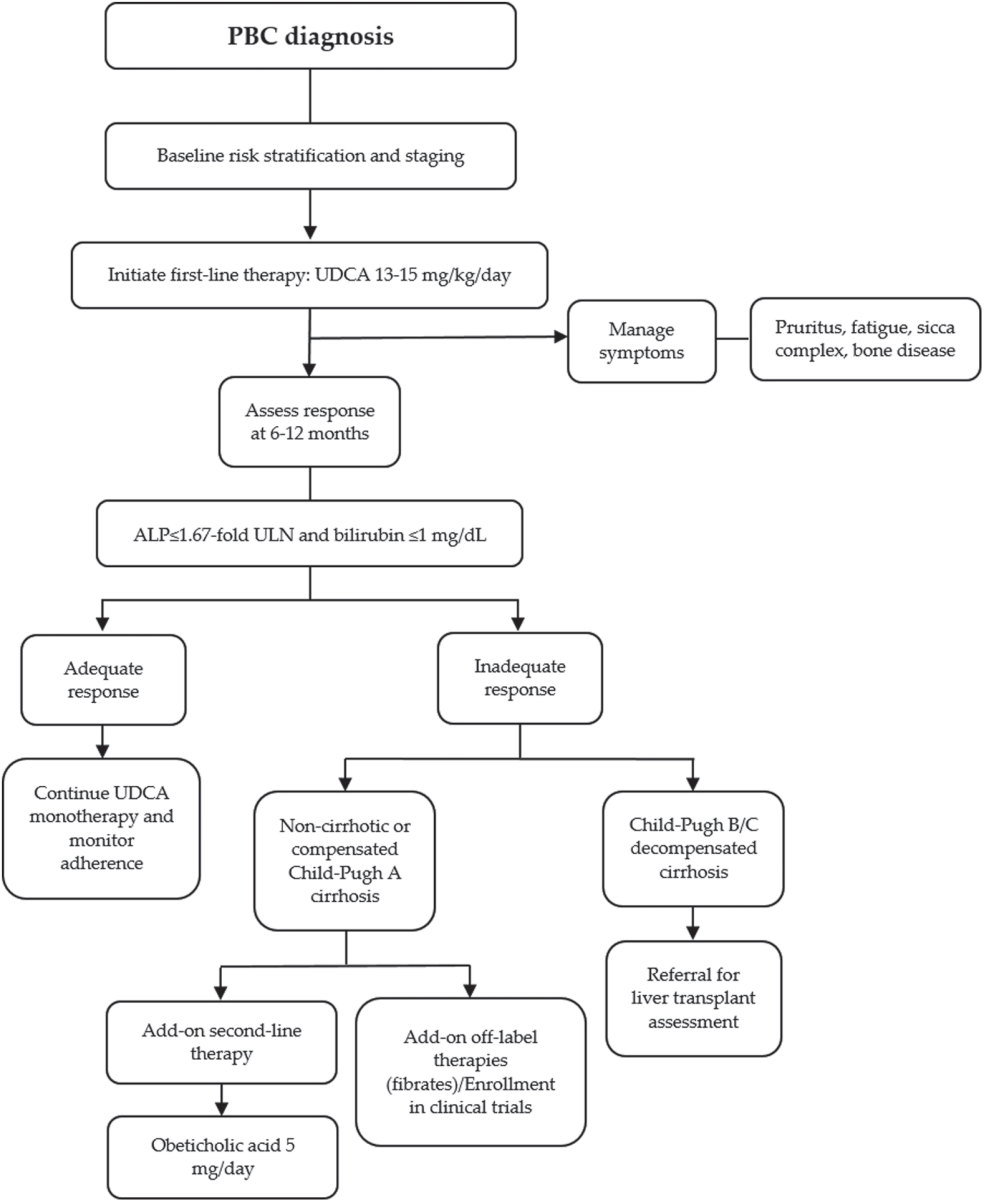
Algorithm for the management of PBC patients with disease-modifying agents. PBC, primary biliary cholangitis; UDCA, ursodeoxycholic acid; ALP, alkaline phosphatase; ULN, upper limit of normal.

Ursodeoxycholic acid constitutes the current first-line therapy for PBC by the American Association for the Study of Liver Diseases and the European Association for the Study of the Liver for all patients since its approval by the FDA back in 1997. Based on current guidelines, UDCA is recommended at a dose of 13-15 mg/kg per day, started progressively, and can be administered as a single oral daily dose or divided doses (Figure 1).^5^

Ursodeoxycholic acid is a hydrophilic dihydroxy bile acid whose oral administration can enrich the bile acid pool and decrease the hepatotoxic effects of hydrophobic bile acids when BA retention occurs.^8^ The role of UDCA in the treatment of PBC has been studied, and multiple hepatoprotective mechanisms have been proposed, including inducing choleresis, antiapoptotic activity, anti-fibrotic, anti-inflammatory, and immunomodulatory properties.^6,8^ Several randomized clinical trials of UDCA have been reported and showed consistent improvement in the liver biochemical profile, IgM levels, and liver histology. In clinical practice, studies have shown that UDCA improves biochemical indices, delays histological progression, improves survival, and decreases the need for liver transplant.

The Global PBC Study Group in 2014 reported results from a cohort of 4854 patients, a 10-year cumulative liver transplant-free survival of 79.7% (95% CI 79.1-81.2) in patients receiving UDCA, compared with 60.7% (95% CI 58.2-63.4) in untreated patients (*P* < .001). Later, in 2020, data were included from 3902 patients with a median follow-up of 7.8 years, showing that UDCA was associated with a statistically significant reduction in the risk of LT or death (hazard ratio [HR] 0.46, 95%CI 0.40-0.52, *P* < .001). Additionally, the overall number of patients with PBC needed to be treated with UDCA to prevent 1 LT or death within 5 years was 11 (95% CI 9 to 13). These results confirmed that UDCA confers a survival benefit for PBC patients and further supports its use for standard medical therapy.^9^

Regarding its safety profile, UDCA is a safe drug at the standard dose in patients with any stage of PBC, with no need for dosage adjustment in patients with other concomitant liver or renal diseases. Associated side effects include weight gain, hair thinning, mild gastrointestinal disorders such as diarrhea, nausea, and vomiting.

Once first-line therapy has started, current guidelines recommend that the need for second-line therapies should be assessed after 1 year of treatment initiation based on biochemical response (Figure 1).^1^ Several criteria have been proposed to assess treatment response and commonly include a decrease in alkaline phosphatase (ALP) with or without bilirubin normalization (Table 1).^6^ Qualitative criteria dichotomize patients into UDCA responders or non-responders, and quantitative scoring systems quantify the subject’s risk of death or liver transplant in relation to time (Table 1). While scoring systems vary, prior studies have shown that risk stratification models, particularly the GLOBE and United Kingdom-primary biliary cholangitis (UK-PBC) risk scoring systems, are quite accurate at predicting adverse events, and are perhaps superior to UDCA treatment response models.^2^

Unfortunately, approximately 40% of patients may not respond to UDCA, and their clinical outcomes are significantly worse than UDCA responders. Early identification of non-responders is essential to anticipate the need for other treatments and prevent complications.^5,6,10^ Patients who are intolerant to UDCA or have an inadequate response at 12 months of treatment should be evaluated for alternate treatment options including second-line (OCA) or investigational therapies.

### Obeticholic acid

Obeticholic acid is FDA approved for the treatment of PBC and is considered second-line therapy. Obeticholic acid is an analog of chenodeoxycholic acid, a bile acid, and additionally selectively activates the farnesoid X receptor (FXR).^11^ Farnesoid X receptor activation through OCA has been associated with lower rates of hepatic fibrosis and inflammation.^11^ It was first shown to offer a benefit to UDCA non-responders by Hirschfield et al^11^ in a phase 2 randomized control trial (RCT), showing significant reductions in alkaline phosphatase at 1 year. This has been further verified and reproduced in subsequent trials, including the POISE trial by Nevens et al in 2016.^12^

Obeticholic acid originally gained FDA approval for UDCA non-responders or for those unable to tolerate UDCA for PBC patients in 2016. However, since then, a black box warning emerged in) May 2021 stating the increased risk of decompensated cirrhosis and liver failure in patients with advanced cirrhosis. It is now contraindicated in patients with decompensated cirrhosis or compensated cirrhosis with evidence of portal hypertension. Additionally, OCA itself can be poorly tolerated and can cause pruritus.

## Investigational Therapies for Disease-Modifying Therapies

### Peroxisome Proliferator-Activated Receptor Agonists

Peroxisome proliferator-activated receptor agonists are a heterogeneous family of nuclear receptors. Agonists targeting these receptors have a wide variety of effects and have been studied for pharmacotherapy for diabetes, hyperlipidemia, liver disease, and pulmonary disease. Peroxisome proliferator-activated receptor agonists exist in 3 main isoforms, alpha, beta/delta, and gamma. Clinically, PPAR-alpha agonists such as fenofibrate have historically been used for dyslipidemia and hypertriglyceridemia. Peroxisome proliferator-activated receptor-gamma agonists such as thiazolidinediones were historically used in the treatment of diabetes mellitus, although their use has fallen out of favor over the last several years due to their side effect profile. With regards to PBC, PPAR agonists are being studied as a potential disease-modifying therapy (DMT) agent, which is discussed further. Additionally, PPAR agonists have been studied for the symptomatic treatment of pruritus, which falls outside the scope of this review.

#### Fibrates

Recent studies have examined the efficacy of fibrates as a DMT for PBC non-responders. The effect of bezafibrate, fenofibrate, and elafibranor have all been studied in PBC patients.

#### Bezafibrate

Bezafibrate is a pan-PPAR receptor agonist, activating all isoforms of the receptor. A systematic review analyzing trials looking at a combination of bezafibrate and UDCA for patients with PBC found significant improvements in liver biochemistry and prognosis estimated by risk calculators, but no improvement in clinical symptoms or mortality.^13^ Subsequently, in the landmark BEZURSO trial, a randomized placebo-controlled trial among UDCA non-responders, 31% of patients in the Bezafibrate group achieved biochemical remission at 24 months.^14^ Furthermore, Soret et al^15^ showed that combination triple therapy with bezafibrate, OCA, and UDCA in non-responders resulted in a significant improvement in alkaline phosphatase and other liver biochemistry markers, compared to dual treatment with OCA and UDCA alone. Bezafibrate appears to assist in inducing biochemical remission in UDCA non-responders and improve prognosis based on predictive models, but further longitudinal studies are needed to assess its impact on hard outcomes including liver transplantation and mortality.

#### Elafibranor

Elafibranor, a dual PPAR-alpha and gamma agonist, has been recently evaluated in patients with PBC. In a double-blind 12-week phase 2 trial including 45 participants with a poor, inadequate response ursodeoxycholic acid, participants were randomized to either Elafibranor 80 mg, Elafibranor 120 mg, or placebo.^16^ The Elafibranor groups (80 mg and 120 mg) achieved the primary endpoint (total bilirubin less than the upper limit of normal, alkaline phosphatase less than 1.67× the upper limit of normal, and a drop in the alkaline phosphatase greater than 15%) in 67% and 79% of cases, respectively.^16^ Further studies with larger sample size and longer treatment duration are required to reinforce these findings, but thus far Elafibranor appears to be a promising option for DMT in UDCA non-responders.

#### Fenofibrate

Fenofibrate is a selective PPAR-alpha agonist that has also been studied in patients with PBC as a potential DMT agent. A pilot study in 2011 looking at the addition of fenofibrate to UDCA non-responders showed a significant improvement in biochemical response at 48 weeks, compared to baseline values.^17^ However, perhaps the most well-known study of fenofibrate in PBC patients was performed by Hegade et al^18^ in 2016, who studied the addition of fenofibrate to UDCA in UDCA non-responders with outcomes available through 3 years of treatment. This study demonstrated that long-term treatment with fenofibrate in addition to UDCA did improve biochemical data including alkaline phosphatase but did not lower the estimated risk of liver-related mortality or need for liver transplant.^18^ This has since been further reproduced in subsequent trials again demonstrating that fenofibrate addition may lower alkaline phosphatase levels but does not offer a clear mortality benefit, and adverse effects such as elevated liver function tests and renal insufficiency may occur.^19^

#### Seladelpar

Seladelpar is a selective PPAR-delta agonist that has been studied as a potential DMT agent in PBC patients. In phase 2 double-blind placebo-controlled trial where patients were assigned to seladelpar at 50 mg, 200 mg, or placebo, patients in the seladelpar group showed normalization of alkaline phosphatase.^20^ Unfortunately, this study was terminated early for safety concerns given asymptomatic elevations of liver function tests.

The ENHANCE study, a phase 3, multicenter, randomized placebo-controlled trial, explored the effect of seladelpar at 5 mg and 10 mg against placebo.^21^ This study showed seladelpar at both 5 mg and 10 mg doses resulted in significantly improved alkaline phosphatase levels and bilirubin levels compared to placebo. However, this study was terminated early after concerning histological findings arose in a different seladelpar trial in patients with non-alcoholic steatohepatitis (NASH). Kremer et al^22^ showed in a 1-year phase 2 study without placebo that seladelpar-treated patients reported significant improvements in serum bile acid levels as well as pruritus and sleep.

Given continued uncertainty regarding the safety of seladelpar and its efficacy as a potential DMT agent, further studies are ongoing to evaluate its potential use in PBC patients. The RESPONSE trial is a randomized, placebo-controlled phase 3 study to assess the efficacy of seladelpar in PBC patients who are non-responders or intolerant of UDCA and is currently underway (Clinical Trials ID: NCT04620733).

#### Saroglitazar

Saroglitazar is a PPAR-alpha/gamma receptor agonist that is being studied for the treatment of PBC. A prior phase 2 proof of concept randomized placebo-controlled trial noted significant reduction of alkaline phosphatase levels at 16 weeks in patients randomized to both the saroglitazar 4 mg and 2 mg groups.^23^ However, patients randomized to the saroglitazar group, particularly in the 4 mg group, experienced higher levels of adverse events, including significantly elevated liver function tests that resolved after drug discontinuation.^23^ A multicenter RCT with placebo to explore the efficacy and safety of saroglitazar at lower doses in PBC patients is planned, with recruitment set to begin in 2022 (Clinical Trials ID: NCT05133336).

### Farnesoid X Receptor Agonists

Bile acids are endogenous FXR agonists that work by activating this nuclear receptor in different cell types, including hepatocytes, and therefore have a role in many metabolic disturbances, including cardiovascular disease, diabetes, obesity, and NAFLD. Its role in PBC in the form of obeticholic acid is thought to be the regulation of the expression of cholesterol 7 alpha-hydroxylase, the rate-limiting enzyme in BA synthesis. It has also been shown to help heal liver injury from cholestasis and steatohepatitis in NAFLD and NASH.

#### ASC42

ASC42 is a potent FXR agonist that was developed as a potential treatment for PBC patients. Similar to the mechanism of action of OCA, ASC42 through stimulation of the FXR is theorized to decrease bile acid synthesis resulting in lower rates of hepatic inflammation and fibrosis.^3^ ASC43 (Clinical Trials ID: NCT05190523) is set to begin recruitment for phase 2 clinical trials early in 2022.

#### Cilofexor

Cilofexor is an FXR agonist which has been shown to improve cholestatic pattern of liver injury and transaminase abnormalities when used for PSC and NASH.^24^ Thus far in humans, cilofexor monotherapy in NASH patients does not appear to significantly mitigate hepatic fibrosis, and some patients develop moderate to severe pruritus during treatment, although perhaps less than that observed with OCA.^24^ A single clinical trial has been NIH funded for its use in PBC but terminated early because of the availability of alternative therapies for PBC (Clinical Trials ID: NCT02943447).

#### Tropifexor

Tropifexor is another FXR agonist first evaluated in animal models for cholestatic liver diseases and NASH.^25^ It has been shown to mitigate hepatic inflammation, steatosis, and reduce gamma-glutamyl transferase (GGT) levels in a dose-dependent manner with correlated increases in fibroblast growth factor-19 (FGF-19) levels. In NASH patients, it has been shown to result in minimal pruritus and other side effects, including stable lipid panels (Clinical Trials ID: NCT02855164). A prior phase 2 placebo-controlled double-blinded study assessing tropifexor in PBC patients showed a statistically significant and dose-dependent reduction in GGT levels in the treatment arm (Clinical Trials ID: NCT02516605), without any serious adverse events occurring. Further studies are needed to confirm these initial findings.

#### EDP-305

The FXR agonist EDP-305 may be more relevant for mitigating the progression of PBC as it has been shown to reduce interstitial renal fibrosis and hepatic fibrosis in NASH.^26,27^ However, a previous clinical trial in PBC patients, the INTREPID trial (NCT03394924), failed to meet the primary endpoint of a 20% reduction in the alkaline phosphatase. Further studies may be warranted to assess if the FXR agonists could serve as a novel disease-modifying drug for PBC.

### Fibroblast Growth Factor-19 Agonists

Fibroblast growth factor 19 is released by enterocytes in a feedback mechanism and works through FGFR4 in hepatocytes to reduce bile acid synthesis, making it a novel therapeutic for PBC.^28^ Fibroblast growth factor-19 analogs are able to mitigate steatosis in the liver by regulating oxidative stress and autophagy.^28^

#### Aldafermin (NGM 282)

The FGF-19 analog aldafermin (NGM 282) has been shown to improve liver fibrosis, which theoretically may reduce the incidence of cirrhosis within this patient population.^28^ In PBC patients who had failed UDCA, NGM 282 over 28 days resulted in reduction of ALP and transaminase levels compared with placebo, with an acceptable safety profile.^29^

### NADPH Oxidase 3 Inhibitors

NADPH oxidase 3 inhibitor is a family of enzymes that has been shown to be involved in hepatic fibrosis through the activation of hepatic stellate cells. They have been developed for potential use in PBC.

#### Setanaxib

Setanaxib, a dual NOX-4 and NOX-1 inhibitor, has shown promising results in an interim analysis from a phase 2 clinical trial. An interim analysis indicated that setanaxib in a 400 mg dose once and twice a day could reduce serum GGT and ALP levels, as well as dose-dependent reductions of liver transaminases and high sensitivity C-reactive protein at 6 weeks of treatment in PBC patients with an inadequate response to UDCA.^30^ The trial has since finished, its final results at 24 weeks are pending publication. A phase 2/3b trial, the TRANSFORM trial is a 52-week, currently in the recruitment phase, will initially investigate the effect of setanaxib at higher doses (1200 mg/day and 1600 mg/day) on ALP reduction in patients with PBC with elevated liver stiffness and intolerance or inadequate response to UDCA.

### Janus Kinase Inhibitors

Baricitinib is a novel Janus Kinase (JAK) inhibitor initially developed for the treatment of Rheumatoid arthritis but has since been also used in the treatment of severe COVID-19 infection. Baricitinib is theorized to downregulate multiple cytokines that may play a role in PBC, including type I Interferon. A clinical trial (NCT03742973) examining the effects of baricitinib in PBC in UDCA non-responders found a significant decline in alkaline phosphatase after a 12-week course of treatment; however, the study was ultimately terminated early due to lack of enrollment (only 1 patient was randomized to the Baricitinib arm). Future studies investigating the effects of JAK inhibitors for UDCA non-responders would be warranted.

### Etrasimod

The sphingosine 1-phosphate receptor (S1PR) plays a variety of roles when activated, including lymphocyte cell trafficking.^31^ Etrasimod was developed as a S1PR agonist, and a previous clinical trial (NCT03155932) looked at etrasimod as an immunomodulator medication for patients with PBC in a phase 2 proof of concept study. While the study was terminated due to sponsor decision, further studies investigating the effect of S1PR agonists may be warranted.

## Conclusion

Primary biliary cholangitis is a challenging disease characterized by progressive destruction of bile ducts and subsequent cholestasis, which can ultimately progress to cirrhosis. Ursodeoxycholic acid is considered first-line therapy. Unfortunately, ~40% of PBC patients have an incomplete response to UDCA and carry a worse prognosis when compared to responders. The need for additional therapies is assessed based on the patient biochemical response after 1 year of UDCA therapy.

Obeticholic acid has been shown to be effective in UDCA non-responders and currently is considered second-line therapy. However, it is contraindicated in cirrhotic patients who have evidence of portal hypertension. There is a great need for additional pharmacotherapy to induce disease remission in PBC patients who do not respond to UDCA. Peroxisome proliferator-activated receptor and FXR agonists are pondered as DMTs since they potentially improve liver fibrosis. Fibroblast growth factor-19 agonists and NOX inhibitors are also under investigation for further DMT options. Evidence supporting one medication over others is sparse, highlighting the need for both additional longitudinal clinical trials, as well as research aimed at understanding the underlying pathophysiology of PBC to discover further therapeutic targets.

## Figures and Tables

**Table 1.** Criteria for Predicting Outcomes in Primary Biliary Cholangitis Patients: Biochemical Response to UDCA and Risk Stratification

Biochemical Response to Treatment			
Qualitative Definition	Number of Patients	Time to Assessment	Responder Criteria
Rochester-I^32^	180	6 months	ALP ≤2-fold ULN
Barcelona*^33^	192	1 year	ALP decreases greater than 40% of baseline values or normal levels after 1 year of treatment.
Paris-I*^6^	291	1 year	Bilirubin level ≤1.0 mg/dL ALP ≤3-fold ULN AST ≤2-fold ULN
Rotterdam^34^	375	1 year	Normalization of bilirubin and/or albumin levels
Toronto*^35^	69	2 years	ALP ≤1.67-fold ULN
Ehime^36^	83	6 months	Normal GGT levels or ≥70% decrease in pre-treatment levels
Paris-II^37^	165	1 year	ALP and AST ≤1.5-fold ULN with a normal bilirubin level
Rochester-II^4^	73	1 year	ALP ≤1.67-fold ULN and bilirubin ≤1 mg/dL
Global PBC^38^	4845	1 year	ALP ≤2-fold ULN and bilirubin ≤1-fold ULN
Risk Stratification Scores			
Quantitative scores	Number of patients	Time to assessment	Included parameters
APRI-r1 (± biochemical response)^39^	386	1 year	Aspartate aminotransferase (AST) and platelet count at 1 year of treatment APRI ≤0.54 ±biochemical response (defined by Barcelona, Paris I/II, or Toronto criteria)
GLOBE score^40^	4119	1 year	Age at start of UDCA ALP, bilirubin, albumin, and platelet count at 12 months
UK-PBC score^41^	3165	1 year	Baseline albumin and platelet count ALP, bilirubin, and AST (or ALT) at 12 months of treatment.

ALBI, albumin-bilirubin; ALP, alkaline phosphatase; ALT, alanine aminotransferase; APRI-r1, AST/platelet ratio index at 1 year of treatment; AST, aspartate aminotransferase; GGT, gamma-glutamyl transferase; PBC, primary biliary cholangitis; ULN, upper limit of normal.

*Considered the best predictability of transplant-free survival as validated in large studies.

**Table 2.** Investigational Therapeutics for the Treatment of Primary Biliary Cholangitis

	Mechanism of Action	Clinical Trial Stage	Dosage		Primary Endpoints	Number of Participants	Final/Preliminary Results	Limitations/Adverse Events
PPAR agonists								
Bezafibrate^16^	Pan-PPAR receptor agonist	Phase 3 (BEZURSO) completed	400 mg/day		Percentage of patients with complete biochemical response at 24 months -Normalization of AST, ALT, ALP, albumin, bilirubin, and prothrombin index.	100	Biochemical response in 31% of bezafibrate. ALP normalization in 67% of the patients in the bezafibrate group.	Creatinine level increased 5% from baseline in the bezafibrate group. Myalgia in 20% of patients in the bezafibrate group.
Fenofibrate^17,18^	PPAR-alpha agonist	Phase 2 completed	160 mg/day		Difference in median ALP at 1 year compared to baseline values.	20	Median serum ALP decreased significantly at 48 weeks from 351 (214-779) U/L at baseline to 177 (60-384) U/L at 48 weeks.	It was an uncontrolled, open-label pilot study. Heartburn was the most frequent adverse event.
Selaldepar^22,42^	PPAR-delta agonist	Phase 3 completed (ENHANCE)	5 mg titrated to 10 mg/day -10 mg/day		Composite response by month 3 that included: ALP <1.67 × ULN, ≥ 15% decrease in ALP, and total bilirubin ≤ ULN	112	Composite response was statistically significantly higher for the 5 mg (78.2%) and 10 mg (57.1%) arms than the rate for the placebo arm.	Early termination due to an unexpected histologic finding in a clinical trial of seladelpar for NASH. Most common adverse events: pruritus and abdominal pain.
		Phase 2 terminated	-Seladelpar/MBX-8025 50 mg -Seladelpar/MBX-8025 200 mg		Percentage change from baseline in ALP at 12 weeks	41	Changes in both seladelpar groups versus placebo were significant (*P* < .0001), no significant difference between seladelpar groups (*P* = ·1729).	Early termination due to increases in aminotransferases associated to treatment.
Elafibranor^18^	PPAR-alpha/delta agonist	Phase 2 completed	-Elafibranor 80 mg -Elafibranor 120 mg		Relative change from baseline is in serum ALP levels at week 12	45	Significant ALP reductions in elafibranor groups compared to placebo. Elafibranor 80 mg: -48.3% Elafibranor 120 mg: -40.6%	Reported adverse events: nausea, diarrhea, fatigue, and headache.
Saroglitazar^23^	PPAR-alpha/gamma agonist	Phase 2 completed (EPICS)	Saroglitazar magnesium 2 mg Saroglitazar magnesium 4 mg		Improvement in ALP levels after 16 weeks	37	Significant mean percentage reductions in ALP in the saroglitazar 4 mg (49%) and 2 mg (51%) groups.	Study drug discontinued in 4 patients from the study group due to aminotransferase increases.
FGF19 analogs								
NGM282^31^	FGF19 analog	Phase 2 and 2b completed	Subcutaneous NGM282 doses of -0.3 mg -3 mg		Absolute change in ALP from baseline to day 28.	45	ALP levels were significantly reduced with NGM282, with LS mean difference from baseline of: -0.3 mg: –54.3 IU/L -3 mg: –69.3 IU/L	No significant change in the proportions of patients achieving ALP normalization or less than 1.67× ULN with NGM282 treatment.
FXR agonists								
Tropifexor (LJN452)^43^	FXR agonist	Phase 2 completed	-30 µg -60 µg -90 µg		Fold change in serum GGT on day 28	61	Interim analysis showed a 72% decrease of GGT in treatment group at 4 weeks	-
Cilofexor (GS-9674)^44^	FXR agonist	Phase 2 terminated	-30 mg -100 mg		Safety and tolerability	71	Reduction ≥ 25% in ALP from baseline to week 12 in 17% on cilofexor 100 mg and 18% on 30 mg.	Pruritus leading to treatment discontinuation occurred in 7% of patients on cilofexor 100 mg
EDP-305	FXR agonist	Phase 2 completed (INTREPID)	-1 mg -2.5 mg		Proportion of patients with ≥ 20% reduction in ALP or ALP normalization at week 12.	68	Reduction ≥ 20% in ALP at week 12 in 45.2% (*P* = .106) on EDP-305 1 mg, 46.4% on 2.5 mg (*P* = .063), compared to 11.1% on placebo.	Treatment discontinuation due to pruritus in 3% for the 1 mg and 18% in the 2.5 mg EDP-305 groups.
ASC42	FXR agonist	Phase 2 ongoing	-5 mg -10 mg -15 mg		Percentage changes of ALP at day 85 compared with baseline.	-	Not yet recruiting	-
Other targets								
Setanaxib (GKT137831, GKT831)	NOX1 and NOX4 inhibitor	Phase 2 completed	-400 mg twice daily 400 mg once daily		The percent change in serum GGT from baseline to week 24.	-	Results pending	Results pending
		Phase 2b/3 (TRANSFORM) recruiting	-1200 mg/day 1600 mg/day		Proportion of patients achieving biochemical response at week 52: ALP <1.67× ULN ALP reduction ≥ 15% from baseline Total bilirubin ≤1 xULN	-	Results pending	Results pending
Mesenchymal stem cells^45^	T-cell suppressant	Phase 1/2 completed	0.5 x 10^6^cells/kg body weights 3 times at 4-week intervals		Serum ALP at weeks 0, 4, 8, 12, 24, 36, and 48 after treatment.	7	Significant decrease in serum ALP at 48 weeks from baseline (474.29 ± 223.26).	Small study population.
		Phase 2 recruiting	0.1-1 x 10^6^ cells/kg body weight 3 times (week 0, 4, and 8)		Absolute change of ALP after 1 year of the initial stem cell treatment	-	Results pending	
Etrasimod (APD334)	S1PR1, S1PR4, and S1PR5 agonist	Proof of concept study terminated			Change from baseline to week 24 in serum ALP.	-	No results available	Early termination due to sponsor decision.
Baricitinib	Janus Kinase (JAK) inhibitor	Proof of concept study terminated	-1 mg -2 mg		Change in ALP at week 12 from baseline	-	No results available	Early termination due to enrollment futility.
E6011	Anti-CX3CL1 antibody	Phase 2 terminated			Rate of ALP change from baseline at week 12.	-	No results available	Early termination due to lack of response after 12 weeks of treatment.
S-adenosyl-L-methionine (SAMe)^46^	Assists with detoxification and prevents oxidative stress	Phase 4	1200 mg daily for 6 months		Changes in PBC-40 questionnaire	24	Significant improvement in PBC-40 fatigue and pruritus, decreases in ALP, GGT, Total cholesterol in non-cirrhotic PBC patients	-
NI-0801	Human monoclonal antibody targeted against chemokine ligand 10 (CXCL10)	Phase 2a	6 infusions at 10 mg/kg		Liver function tests	29	No significant reduction in liver function tests	Headaches, fatigue, pruritus, diarrhea
Combined Antiretroviral Therapy (cART): Tenofovir-Emtricitabine (TDF/FTC), and Lopinavir-Ritonavir (LPRr)	Anti-retroviral therapy	Phase 2	TDF/FTC (300/200 mg daily) and LPRr (400/100 mg twice daily)		Reduction in alkaline phosphatase below 1.67 x the upper limit of normal, or normalization of total bilirubin	13	Significant improvement alkaline phosphatase but failure to meet the primary endpoint	Poorly tolerated due to significant gastrointestinal side effects
Pentoxifylline	Inhibits pro-inflammatory cytokines and anti-fibrotic effects	Pilot Study completed	400 mg 3 times daily		Change in serum ALP from baseline to 6 months	20	ALP levels were significantly reduced at month 6 (–57.3 IU/L) when compared to baseline.	It was a small open-label pilot.
Probiotics	Regulation of bile acid homeostasis	Phase 2 Not yet recruiting	One pack 3 times per day		Percentage of patients with ALP or GGT decreased by 20% from baseline at 6 months	-	Not yet recruiting	-
OP-724	Beta-catenin inhibitor	Phase 1 (NCT04047160)	140, 280, 380 mg/m^2^/4 hours twice a week		Occurrence of serious adverse events	12	Trial is ongoing	

ALP, alkaline phosphatase; ALT, alanine aminotransferase; AST, aspartate aminotransferase; FGF19, fibroblast growth factor 19; FXR, farnesoid X receptor; GGT, gamma-glutamyl transferase; NASH, non-alcoholic steatohepatitis; NOX, nicotinamide adenine dinucleotide phosphate oxidase; PBC, primary biliary cholangitis; PPAR, peroxisome proliferator-activated receptor; S1PR, sphingosine 1-phosphate receptors; ULN, upper limit of normal.
